# Mitogenomic characterization and phylogeny of *Scelimena melli* Günther (Orthoptera: Tetrigoidea: Scelimeninae)

**DOI:** 10.1080/23802359.2021.1978887

**Published:** 2021-09-22

**Authors:** Xuejuan Li, Chuhui Lin, Lenan Wang, Zichen Gao, Tingting Zhu, Liliang Lin

**Affiliations:** College of Life Sciences, Shaanxi Normal University, Xi’an, China

**Keywords:** *Scelimena melli*, Tetrigoidea, mitogenome, phylogeny

## Abstract

The mitochondrial genome (mitogenome) of *Scelimena melli*, which belongs to Orthoptera, Tetrigoidea, Tetrigidae, Scelimeninae was determined. The mitogenome has a length of 14,598 bp and consists of 37 genes including 13 protein-coding genes (PCGs), two rRNA genes, and 22 tRNA genes. Phylogenetic analysis using 37 mitochondrial genes with other 22 Tetrigoidea species revealed that *S. melli* had a closer relationship with *Paragavialidium sichuanense*, but the monophyly of Scelimeninae was not recovered. The mitogenome data of *S. melli* would provide useful resources for further evolutionary studies of Scelimeninae and Tetrigoidea species.

Nowadays, mitochondrial genomes (mitogenomes) and transcriptome data were used for exploring the evolution issues of Orthoptera insects (Chang et al. 2020; Song et al. 2020). However, the mitogenome data of Tetrigoidea within Orthoptera of public databases are limited, which restricts their phylogenetic researches. The genus *Scelimena* Serville, 1838 belongs to Scelimeninae, Tetrigidae, Tetrigoidea, Caelifera, and Orthoptera, based on the Orthoptera Species File (OSF) v5.0. This genus currently includes 23 known species in the world, with nine of them distributed in southern China (Deng 2016). Among them, *Scelimena melli* Günther, 1838 has a distribution of Guangxi, Guizhou, Guangdong, and Sichuan (Deng 2016). In this study, the mitogenome of *S. melli* (GenBank accession No. MW722938) was determined using the next-generation sequencing method, and the phylogenetic relationship was analyzed by combining with other 22 Tetrigoidea species.

The *S. melli* specimen was collected from Shiwan Mountain, Guangxi, China in 2012, with coordinates of N21.81°, E107.95°. The specimen was stored at Shaanxi Normal University, Xi’an, China, with the voucher number ET1001 (Url: http://lifesci.snnu.edu.cn/#; Contact person: Liliang Lin, ll_lin@163.com). Total genomic DNA was extracted by DNeasy kit and deposited in College of Life Science (accession number: DNA-ET1001), Shaanxi Normal University. Mitochondrial DNA was sequenced using Illumina Hiseq 2000 by Genesky Biotechnologies Inc., Shanghai, China. The data were assembled with MitoZ 2.4 (Meng et al. 2019), then were annotated using the results of MitoZ software and tRNAscan-SE 1.21 (Lowe and Eddy 1997), with compared to other related Tetrigoidea species.

The mitogenome of *S. melli* is 14,598 bp in length and includes 37 genes (13 PCGs, two rRNAs, and 22 tRNAs). The gene order is similar to that of other Tetrigoidea species (Sun et al. 2017; Chang et al. 2020). The overall base composition is 40.7% A, 20.2% C, 11.6% G, and 27.5% T, with an A + T content of 68.2%, which was relatively lower than that of some other Tetrigoidea species (Lin et al. 2017). For PCGs, the start codon begins with ATA in nad4L, ATC in nad2 and cox1, ATT in nad1 and nad3, TTG in nad6, and ATG in other genes. The stop codon is TAG (nad3 and cytb), T (nad5), and TAA of other PCGs. For rRNAs, the rrnL gene (1296 bp) and rrnS (765 bp) are separated by trnV. For tRNAs, the lengths are varied from 62 bp in trnD, trnG, trnR, and trnF to 70 bp in trnK.

The phylogenetic tree was constructed using the newly sequenced mitogenome (*S. melli*) and 22 other available Tetrigoidea species, with *Pseudothericles compressifrons* (Eumastacoidea) as an outgroup. The phylogeny employed a dataset containing 37 mitochondrial genes and was implemented using the maximum-likelihood (ML) method in IQ-TREE v1.6.12 (Nguyen et al. 2015), with 1000 bootstrap replicates. The phylogenetic relationship didn’t recover the monophyly of Scelimeninae, and this non-monophyly was also supported by previous studies (Chang et al. 2020; Li et al. 2021). Among Scelimeninae, the phylogeny of *S. melli*, *Paragavialidium sichuanense*, *Falconius longicornis* was supported with bootstrap replicate support (BS) = 100 (Figure 1), which phylogenetic relationship was consistent with previous studies (Li et al. 2021). This mitogenome of *S. melli* would provide an important resource for further understanding the mitogenome features of Scelimeninae and Tetrigoidea species, and exploring their taxonomic status.

**Figure 1.**
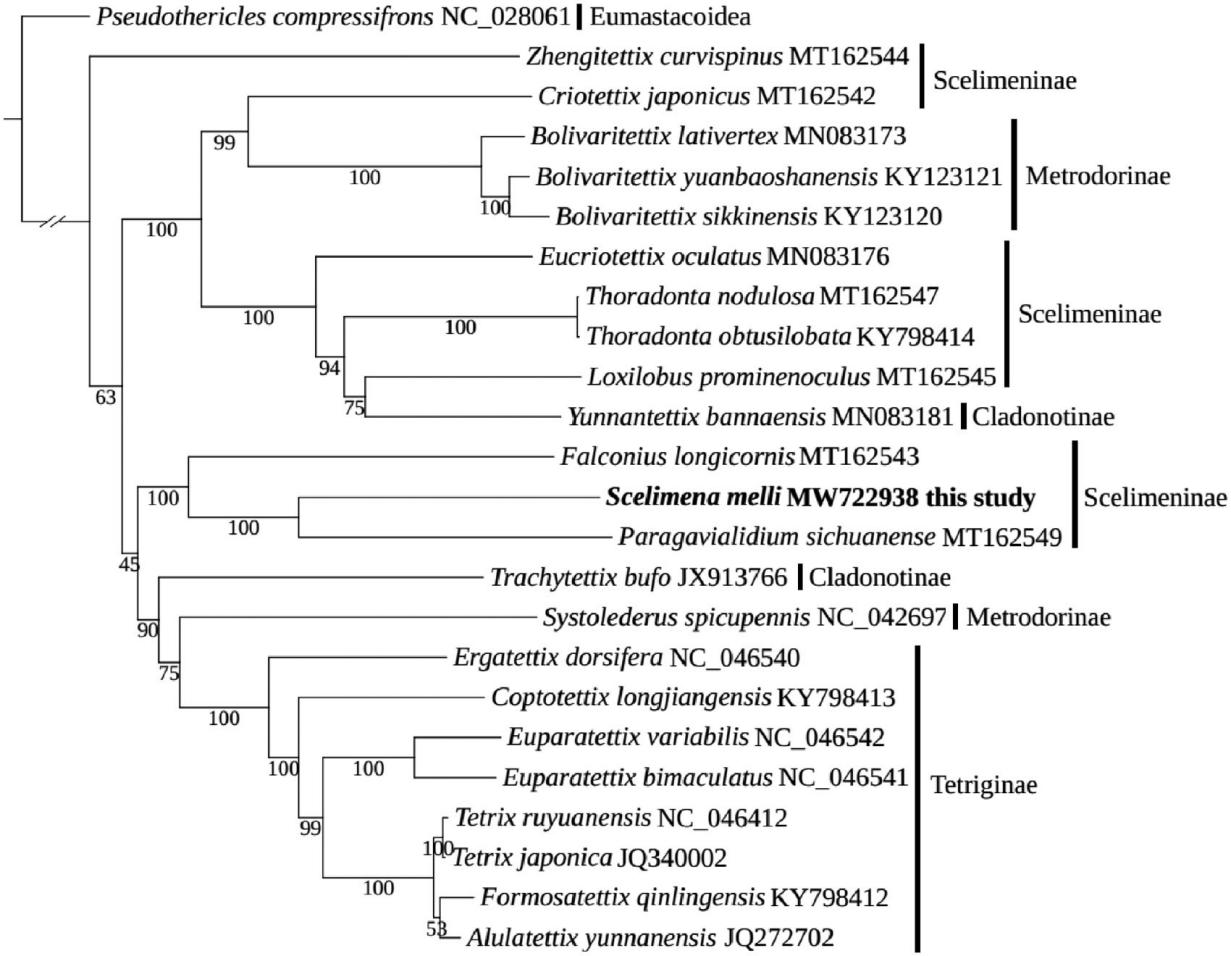
The phylogenetic tree of 23 Tetrigoidea species was reconstructed using mitochondrial genes.

## Data Availability

The mitogenome data of *Scelimena melli* Günther is openly available in the GenBank at https://www.ncbi.nlm.nih.gov/nuccore, with accession No. MW722938. The associated BioProject accession number, SRA data, and BioSample accession number are PRJNA746772, SRR15146522, and SAMN20243815, respectively.

## References

[CIT0001] Chang H, Qiu Z, Yuan H, Wang X, Li X, Sun H, Guo X, Lu Y, Feng X, Majid M, et al. 2020. Evolutionary rates of and selective constraints on the mitochondrial genomes of Orthoptera insects with different wing types. Mol Phylogenet Evol. 145:106734. 31972240 10.1016/j.ympev.2020.106734

[CIT0002] Deng WA. 2016. Taxonomic study of Tetrigoidea from China [Ph.D. dissertation]. Wuhan: Huazhong Agricultural University.

[CIT0003] Li R, Ying X, Deng W, Rong W, Li X. 2021. Mitochondrial genomes of eight Scelimeninae species (Orthoptera) and their phylogenetic implications within Tetrigoidea. PeerJ. 9:e10523. 33604160 10.7717/peerj.10523 PMC7863789

[CIT0004] Lin LL, Li XJ, Zhang HL, Zheng ZM. 2017. Mitochondrial genomes of three Tetrigoidea species and phylogeny of Tetrigoidea. PeerJ. 5:e4002. 29158966 10.7717/peerj.4002 PMC5694214

[CIT0005] Lowe TM, Eddy SR. 1997. tRNAscan-SE: a program for improved detection of transfer RNA genes in genomic sequence. Nucleic Acids Res. 25(5):955–964. 9023104 10.1093/nar/25.5.955 PMC146525

[CIT0006] Meng G, Li Y, Yang C, Liu S. 2019. MitoZ: a toolkit for animal mitochondrial genome assembly, annotation and visualization. Nucleic Acids Res. 47(11):e63. 30864657 10.1093/nar/gkz173 PMC6582343

[CIT0007] Nguyen L-T, Schmidt HA, von Haeseler A, Minh BQ. 2015. IQ-TREE: a fast and effective stochastic algorithm for estimating maximum-likelihood phylogenies. Mol Biol Evol. 32(1):268–274. 25371430 10.1093/molbev/msu300 PMC4271533

[CIT0008] Song H, Béthoux O, Shin S, Donath A, Letsch H, Liu S, McKenna DD, Meng G, Misof B, Podsiadlowski L, et al. 2020. Phylogenomic analysis sheds light on the evolutionary pathways towards acoustic communication in Orthoptera. Nat Commun. 11(1):4939. 33009390 10.1038/s41467-020-18739-4 PMC7532154

[CIT0009] Sun Y, Liu D, Xiao B, Jiang G. 2017. The comparative mitogenomics and phylogenetics of the two grouse-grasshoppers (Insecta, Orthoptera, Tetrigoidea). Biol Res. 50(1):34. 28982393 10.1186/s40659-017-0132-9 PMC5629798

